# Histamine Intolerance: The Current State of the Art

**DOI:** 10.3390/biom10081181

**Published:** 2020-08-14

**Authors:** Oriol Comas-Basté, Sònia Sánchez-Pérez, Maria Teresa Veciana-Nogués, Mariluz Latorre-Moratalla, María del Carmen Vidal-Carou

**Affiliations:** 1 Departament de Nutrició, Ciències de l’Alimentació i Gastronomia, Facultat de Farmàcia i Ciències de l’Alimentació, Campus de l’Alimentació de Torribera, Universitat de Barcelona, Av. Prat de la Riba 171, 08921 Santa Coloma de Gramenet, Spain; oriolcomas@ub.edu (O.C.-B.); soniasanchezperez@ub.edu (S.S.-P.); veciana@ub.edu (M.T.V.-N.); mariluzlatorre@ub.edu (M.L.-M.); 2 Institut de Recerca en Nutrició i Seguretat Alimentària (INSA·UB), Universitat de Barcelona, Av. Prat de la Riba 171, 08921 Santa Coloma de Gramenet, Spain; 3 Xarxa de Referència en Tecnologia dels Aliments de la Generalitat de Catalunya (XaRTA), C/Baldiri Reixac 4, 08028 Barcelona, Spain

**Keywords:** histamine, food intolerance, histamine intolerance, histaminosis, histamine intoxication, diamine oxidase (DAO), low-histamine diet, food supplement

## Abstract

Histamine intolerance, also referred to as enteral histaminosis or sensitivity to dietary histamine, is a disorder associated with an impaired ability to metabolize ingested histamine that was described at the beginning of the 21st century. Although interest in histamine intolerance has considerably grown in recent years, more scientific evidence is still required to help define, diagnose and clinically manage this condition. This article will provide an updated review on histamine intolerance, mainly focusing on its etiology and the existing diagnostic and treatment strategies. In this work, a glance on histamine intoxication will also be provided, as well as the analysis of some uncertainties historically associated to histamine intoxication outbreaks that may be better explained by the existence of interindividual susceptibility to ingested histamine.

## 1. Introduction

In 2011, the European Food Safety Authority (EFSA) issued a scientific report warning that the levels of biogenic amines found in foods marketed in European Union countries may still entail a consumer health risk [1]. Among them, histamine has the highest toxic potential, along with tyramine, and is therefore of great interest in terms of food safety. First described more than 60 years ago, the deleterious effects of excessive histamine ingestion were initially referred to as scombroid fish poisoning or scombrotoxicosis, as they were associated with the consumption of fish in this family, but the condition is now known as histamine intoxication or histamine poisoning. In recent years, another disorder associated with histamine intake, arising from an enzymatic deficiency, has been described. The inability of certain individuals to metabolize histamine in the intestine, resulting in sensitivity to normal or even low histamine levels in food, may help to explain some of the uncertainties historically associated with histamine intoxication.

During the last decade, histamine intolerance has gained social and scientific recognition, with a significant increase in the interest of researchers to investigate this disorder. This review aims to analyze the pathophysiological relevance of dietary histamine, giving special focus to the adverse effects derived from histamine intake and, in particular, to the state of the art concerning the etiology, diagnosis and treatment of histamine intolerance.

## 2. Histamine

**Figure 1:**
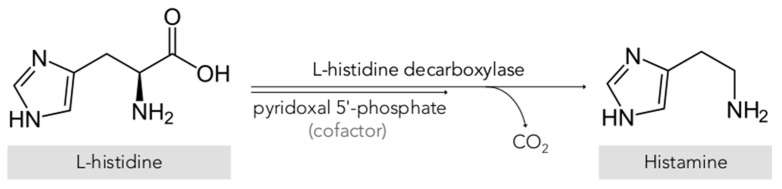
Synthesis of histamine by decarboxylation of its precursor amino acid.

Histamine (2-[4-imidazolyl]ethylamine) is a bioactive amine that is synthesized by decarboxylation of its precursor amino acid, histidine, in an enzymatic reaction first described by Windaus and Vogt in 1907 involving L-histidine decarboxylase (EC 4.1.1.22) (Figure 1) [2]. Due to its chemical structure and number of functional groups, histamine can be defined as a heterocyclic diamine with an imidazole ring and ethylamine (i.e., an organic compound that provides a functional group in the form of a primary amine) [1,3].

The physiological and pathophysiological effects of histamine on the body were first described in 1910 by Dale and Laidlaw, two pioneering researchers who studied the functions of this organic compound at the Wellcome Physiological Research Laboratories [4,5,6]. Specifically, histamine is synthesized and stored in high concentrations in secretory granules, mainly in basophils and mast cells, and also in gastric enterochromaffin cells, lymph nodes and the thymus [1,7]. Functionally, this amine is involved in various immune and physiological mechanisms, stimulating gastric acid secretion, inflammation, smooth muscle cell contraction, vasodilation and cytokine production, among other processes [8,9,10,11]. In addition, histamine functions as a neurotransmitter, being synthesized by neurons located in the posterior region of the hypothalamus whose axons extend through the brain [12]. These wide-ranging physiological effects occur by interaction with four G-protein-coupled receptors with seven transmembrane domains (H1, H2, H3 and H4), which activate signal transduction pathways upon perceiving their ligand, histamine [7,12].

**Figure 2:**
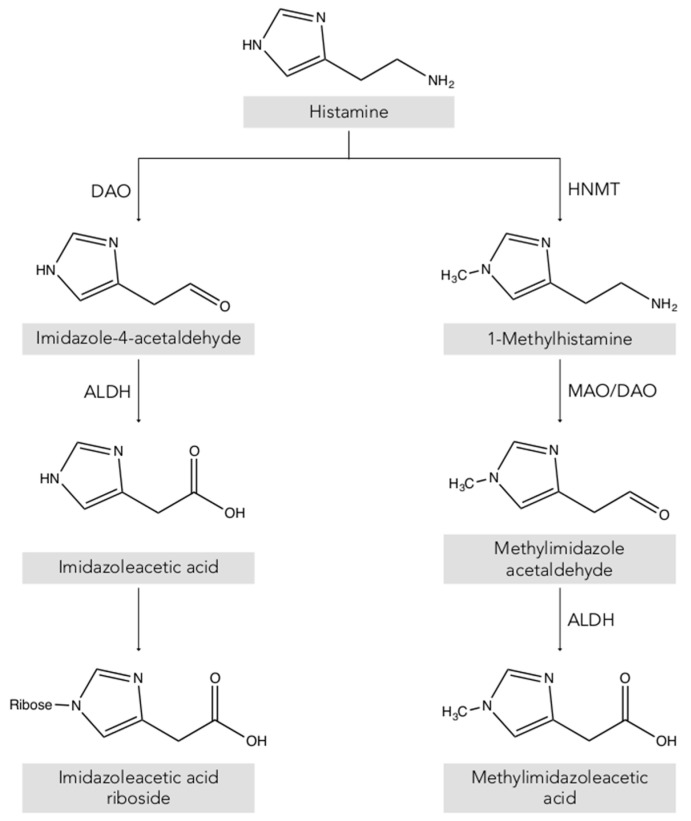
Histamine metabolism in humans. DAO: diamine oxidase; HNMT: histamine-N-methyltransferase; ALDH: aldehyde dehydrogenase; MAO: monoamine oxidase.

Two main histamine metabolic pathways are known in humans, involving the enzymes diamine oxidase (DAO) and histamine-N-methyltransferase (HNMT) (Figure 2) [10,11,13]. DAO (EC 1.4.3.22), also called histaminase or amiloride-binding protein, is a copper-dependent amino oxidase encoded by the AOC1 gene located on chromosome 7 (7q34-36) [14,15,16]. This functional enzyme, a homodimer with two isoforms, catalyzes the oxidative deamination of the primary amine group of histamine [14,16,17]. On the other hand, histamine can be metabolized to 1-methylhistamine by the enzyme HNMT (EC 2.1.1.8), a small monomeric protein encoded by a gene located on chromosome 2q22.1 [18]. HNMT catalyzes the methylation of the secondary amine group of the histamine imidazole aromatic heterocycle by a reaction requiring the S-adenosyl methionine cosubstrate as a methyl group donor [11,13,19].

Thus, depending on its location, the histamine present in the body is deaminated or methylated by the action of the enzymes DAO and HNMT, respectively [1,10,20]. DAO is a secretory protein stored in vesicular structures of the plasma membrane and is responsible for the degradation of extracellular histamine [1,15]. In mammals, the expression of DAO is restricted to certain tissues, mainly the small intestine, ascending colon, placenta and kidneys [14,21]. In the intestine, DAO activity increases progressively from the duodenum to the ileum and is located mainly in the intestinal villi [22]. In contrast, the enzyme HNMT is expressed in a wide range of human tissues, above all in the kidneys and liver, and also the spleen, colon, prostate, ovaries, spinal cord cells and the trachea and respiratory tract [10,13]. HNMT is a cytosolic protein responsible for the inactivation of intracellular histamine and can be synthesized in the cell itself or incorporated from the extracellular space by binding to a receptor or by membrane transporters [7,18]. Regarding substrates, HNMT is highly selective for histamine, whereas DAO can also metabolize other biogenic amines such as putrescine and cadaverine, although it shows a preference for histamine [14,16,23]. The affinity of DAO and HNMT for histamine is very similar, although the latter shows a slightly lower Michaelis–Menten enzymatic constant (K_M_: 6–13 μmol/L) than DAO (K_M_: 20 μmol/L) [10].

The gateway for dietary histamine in the body is the intestinal epithelium. Therefore, although HNMT is also present in the gastrointestinal tract, the more highly expressed DAO plays the major role in protecting the body against exogenous histamine, whether originating from ingested food or generated by the intestinal microbiota [24,25,26]. The protective effect of DAO has been demonstrated in animal experimentation models that were administered aminoguanidine for irreversible and selective DAO inhibition, followed by a dose of histamine [24,27,28]. The development of anaphylaxis symptoms in DAO-inhibited pigs and sheep compared to control groups indicates that the enzyme exerts a significant barrier effect against the absorption of exogenous histamine into the systemic circulation [1,13,19,24,29]. The HNMT enzyme ranks second to DAO in protecting against the absorption of dietary histamine from the intestinal lumen, but appears to be more effective against intravenously or intradermally supplied histamine [13,30].

## 3. Histamine in Foods

Histamine is present in a wide range of foods in highly variable concentrations, which are the main exogenous source of this compound [31]. The main route for histamine formation in food is the decarboxylation of histidine through the action of L-histidine decarboxylase, an enzyme of bacterial origin [32,33]. Apart from histamine, food can also contain other biogenic amines, mainly tyramine (4-hydroxy-phenethylamine), putrescine (1,4-diaminobutane) and cadaverine (1,5-diaminopentane), which are formed through enzymatic deamination of the amino acids tyrosine, ornithine (and/or agmatine) and lysine, respectively [31,34]. The accumulation of these compounds in food is the result of the transformation of amino acids by microorganisms and depends on various factors, such as the availability of the precursor amino acids and environmental conditions favorable for growth and/or the bacterial decarboxylase activity [31,34,35].

These decarboxylation reactions have been described as a survival strategy for microorganisms in acidic environments, as well as an alternative source of metabolic energy in situations of suboptimal substrate availability [1,9]. This enzymatic activity in bacteria is a species- and strain-dependent property [32]. Several Gram-positive and Gram-negative bacteria responsible for microbial spoilage or fermentative processes in food are able to produce histamine [1,36]. Specifically, the Enterobacteriaceae species *Hafnai aluei*, *Morganella morganii* and *Klebsiella pneumonia* have been identified as some of the most prolific histamine-forming bacteria in fish [9,37]. On the other hand, in cheeses, fermented meat, vegetable derivatives and fermented beverages, various lactic acid bacteria have also been described as histamine-producing microorganisms (e.g., *Lactobacillus hilgardii*, *Lactobacillus buchnerii*, *Lactobacillus curvatus* and *Oenococcus oeni*) as well as certain strains of Enterobacteriaceae [1,38,39].

Foods that potentially contain high levels of histamine are: a) those microbiologically altered, such as fish and meat, or derived products that may have been preserved or processed in unsuitably hygienic conditions; and b) fermented products, in which the bacteria responsible for the fermentation process may also have aminogenic capacity [3,40]. Table 1 shows histamine content in the different food categories from the Spanish market [31].

## 4. Uncertainties Associated with Histamine Poisoning: A Paradigm Shift Towards Histamine Intolerance

Although histamine has important physiological functions in the body, it can pose a health risk when ingested in high levels [41]. The proper functioning of histamine degradation systems is key in preventing its accumulation. Histamine intoxication, a kind of food poisoning, may occur after the consumption of foods with an unusually high histamine content that overpowers the degradation mechanisms (generally higher than 500 mg/kg) [1,3,42].

Historically, histamine intoxication has also been termed scombroid fish poisoning or the mahi-mahi flush because of its repeated association with the consumption of fish in the Scombridae and Scomberesocidae families (e.g., tuna, herring and mackerel) [43]. Histamine was first identified in 1946 as the causative agent of the toxic effects of consuming poorly transported tuna, and for a long time histamine poisoning was associated almost exclusively with the consumption of spoiled fish [44,45]. Over the years, the World Health Organization (WHO) has recommended the use of the term histamine intoxication to better designate this pathology, as it can be caused by marine species from other families (e.g., Clupeidae, Engraulidae, Coriphaenidae and Pomatomidae) and even other foods, such as cheese [43]. A meta-analysis carried out in 2018 of the different scientific reports of histamine intoxication between 1959 and 2013 established that the causative food in 98% of cases was fish, the remaining percentage being attributed to cheese [46]. Currently, international health administrations consider histamine intoxication to be one of the main problems of global food security, both for its effects on human health and its impact on trade [47,48].

Histamine intoxication is characterized by occurring in outbreaks and having a short incubation period (i.e., 20–30 min post-ingestion), with symptoms that are generally of low/moderate severity and remit in a few hours [3]. The symptoms are closely linked to the various physiological functions of histamine in the body, affecting the skin (e.g., redness, rash, urticaria, pruritus, edema and local inflammation), the gastrointestinal tract (e.g., nausea, vomiting and diarrhea) and the hemodynamic (hypotension) and neurological (e.g., headache, palpitations and tingling) bodily functions [1,41]. The symptomatic similarity of histamine intoxication with allergy means it is likely to be underdiagnosed [43,48,49]. The diagnosis of histamine intoxication is based primarily on the determination of elevated plasma histamine levels and/or the identification of an ingested food with an unusually high histamine content [13]. In general, an outbreak of histamine poisoning tends to involve more than one individual, lasts a short period of time and a particular causative food is identified [38].

In terms of incidence, the data available for the European Union shows an increase in histamine intoxication outbreaks in the last ten years, unlike other types of food poisoning, and with an almost hegemonic predominance of fish as the causative agent (over 90% of cases) [42,50]. The most recent data from the EFSA and European Center for Disease Prevention and Control (ECDC) show that in 2017, there was a 22% increase in outbreaks compared to the previous year [50]. Specifically, in 2017, there were a total of 117 outbreaks of histamine intoxication involving 572 people, 9% of whom required hospitalization. Fortunately, no deaths have been attributed to histamine poisoning over the past decade [42]. The same trend is observed in the information provided by the European Union Food and Feed Warning System (RASFF), with a progressive rise in the number of cases of histamine poisoning linked to tuna consumption in 2014–2017 and a particularly high increase in 2017 [3].

Although histamine intoxication has been extensively studied in recent decades, unresolved questions remain, concerning, for example, the variable histamine concentrations in the foods triggering outbreaks, or the heterogeneity in the degree and type of adverse effects [46]. Furthermore, the fact that oral administration of histamine in doses equivalent to those normally found in foods causing illness does not produce the same range and/or severity of symptoms is a paradox that has led to multiple hypotheses [30].

Several authors have proposed that alcohol and certain food components, such as other biogenic amines, may have a potentiating effect on histamine toxicity [13,48]. Amines such as putrescine and cadaverine, which are usually found in foods along with histamine, can also act as DAO substrates. It has therefore been suggested that these amines could weaken the protective barrier against dietary histamine by competitively interacting with degradation enzymes in the intestine [3,49]. Other possible potentiators are alcohol and its metabolite acetaldehyde, as they compete with histamine for the enzyme aldehyde dehydrogenase (ALDH), which is simultaneously involved in histamine and alcohol metabolism [1,32]. The potentiation effect of these components could help explain the differences in absorption of the same dose of histamine when ingested in isolation or in a food matrix [48,49]. The FAO and WHO have acknowledged that the involvement of potentiators can alter the threshold dose for toxicity, and they recommend that future studies focus on clarifying the ambiguities in the pathogenesis of histamine intoxication [30].

**Figure 3:**
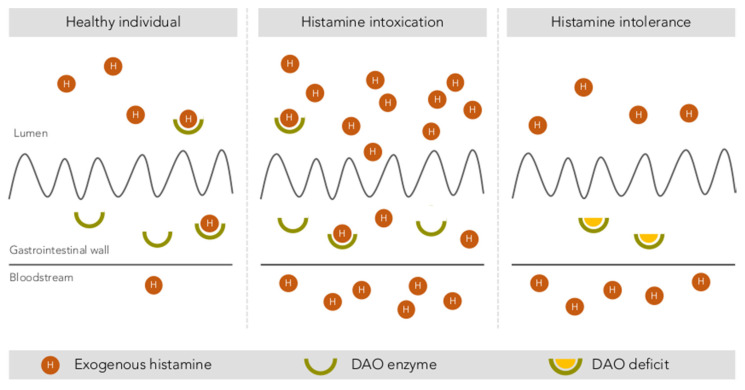
Intestinal degradation of histamine by the DAO enzyme in three different situations: in a healthy individual, with histamine intoxication and with histamine intolerance. Adapted from [13].

Finally, several authors have reported considerable interindividual variability in histamine tolerance, which has been demonstrated in intervention studies [1,3,10,13]. After the oral administration of the same histamine dosage, not all participants showed symptoms, and those who did varied in symptom type and severity and even had different blood histamine levels [48,51,52]. These results indicate the existence of population subgroups with greater sensitivity and clinical responses to histamine, likely linked to a diminished histamine degradation capacity, which could explain some of the historical uncertainties associated with histamine intoxication outbreaks [1]. Without disputing the clinical entity of histamine intoxication, the paradigm shift lies precisely in moving the focus from food to the human body, maintaining histamine as the causative agent, but focusing on how each person is able to respond to the intake of variable levels of histamine from food. Thus, histamine intolerance is the clinical condition that describes the inability of certain individuals to degrade histamine and results in the onset of symptoms caused by its accumulation in the blood (Figure 3).

## 5. Histamine Intolerance

According to the 2003 review of allergy nomenclature by the World Allergy Organization, adverse reactions to food without an immunological basis should be referred to as nonallergic food hypersensitivity, in order to clearly differentiate them from food allergies initiated by a specific immune mechanism [53]. Nonallergic food hypersensitivity is commonly known as food intolerance, a response triggered by a food or any of its components at a dose normally tolerated by the healthy population [54]. While the prevalence of food allergies is estimated at 1–2% in adults, currently almost 20% of the Westernized world’s population suffers from some type of food intolerance, with lactose intolerance being the most common [54].

Histamine intolerance, also referred to as enteral histaminosis or sensitivity to dietary histamine, can be defined as a disorder arising from reduced histamine degradation capacity in the intestine due to impaired DAO activity, leading to its accumulation in plasma and the appearance of adverse effects [11,41,55].

**Figure 4:**
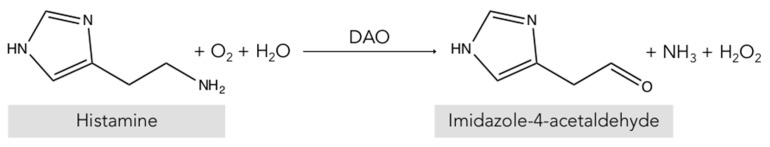
Oxidative deamination of histamine by the DAO enzyme.

The DAO enzyme was first identified back in 1929 by Charles H. Best in autolyzing lung tissue, which he called histaminase because of its ability to degrade histamine [56]. Years later, given its ability to also degrade other diamines, as described above, the more accurate designation of DAO was proposed [57,58]. Beyond its role in the intestinal degradation of histamine in humans, DAO is also present in microorganisms, plants and animals, where it also catalyzes the oxidative deamination of the primary amino group of histamine into its corresponding aldehyde, concomitantly producing stoichiometric amounts of ammonia and hydrogen peroxide (Figure 4) [14,59,60].

**Figure 5:**
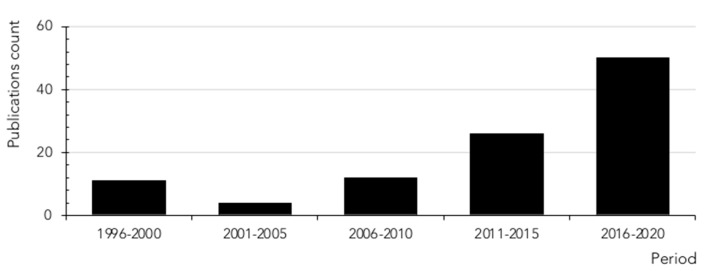
Count of scientific publications containing the keywords histamine intolerance or histaminosis, according to a search performed through the PubMed search engine at the MEDLINE bibliographic database (search performed in July 2020).

Although the first scientific references to histamine intolerance date from more than 20 years ago, it is significant that almost 80% are from the last decade, reflecting the growing interest of researchers in this disorder (Figure 5). In 2011, EFSA already considered histamine intolerance as one of the risks associated with histamine intake, clinically differentiating it from histamine intoxication [1]. In a subsequent joint report, the WHO and FAO emphasized that the no observed adverse effect level (NOAEL) established for histamine was only valid for healthy people, and not for members of susceptible populations, such as those with histamine intolerance [30]. EFSA concluded that only foods with histamine levels below the detection limits are safe for individuals with histamine intolerance [1].

**Figure 6:**
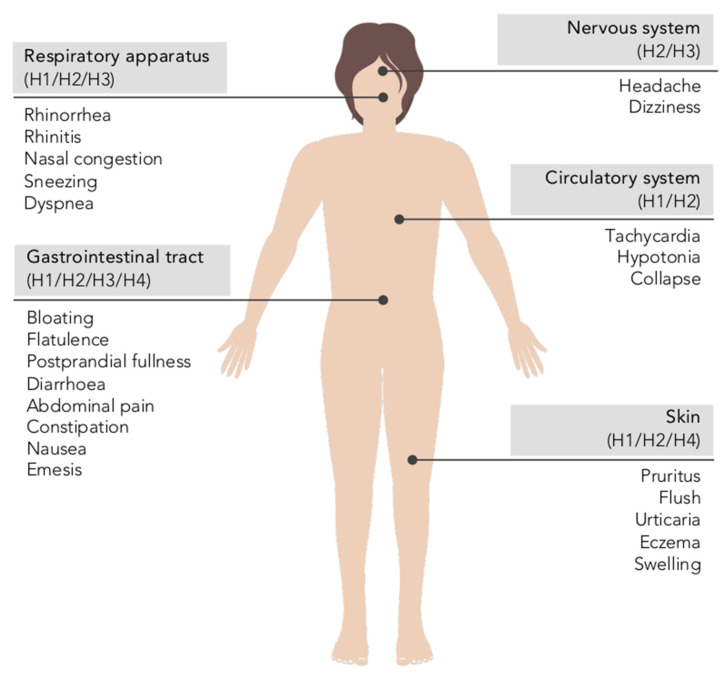
Main symptoms of histamine intolerance and possibly corresponding histamine receptors [10,64].

Clinical manifestations of histamine intolerance consist of a wide range of nonspecific gastrointestinal and extraintestinal symptoms, due to the ubiquitous distribution of the four histamine receptors in different organs and tissues of the body (Figure 6) [10,13,54,61]. In a very recently published study, a team of Austrian researchers comprehensively analyzed the symptoms experienced by 133 patients diagnosed with histamine intolerance [62]. The most frequent and severe manifestations were gastrointestinal, with abdominal distension observed in 92% of patients and postprandial fullness, diarrhea, abdominal pain and constipation in 55–73%. Impairments of the nervous and cardiovascular systems, such as dizziness, headaches and palpitations, were recorded in second place, followed by respiratory and dermatological symptoms. Highlighting the complexity of the clinical picture of histamine intolerance, combinations of three or more symptoms involving different organs were recorded in 97% of cases, with an average of 11 symptoms per patient. The low specificity and complex variability of symptoms undoubtedly contribute to the current difficulty in achieving consensus on the diagnostic criteria for histamine intolerance, as will be discussed in detail below [13]. A lack of data also makes it difficult to determine the current incidence of this condition, although some authors have estimated that it affects 1–3% of the population, a percentage that will possibly increase as more knowledge and diagnostic tools for histamine intolerance become available [10,13,63].

### 5.1. The Etiology of Histamine Intolerance

As mentioned in previous sections, the main barrier against exogenous histamine in the intestines is the DAO enzyme, which prevents its passage into the systemic circulation [10,13,65]. Numerous clinical studies have provided data on the prevalence of low plasma DAO levels in individuals showing symptoms of histamine intolerance, mainly headaches and gastrointestinal or dermatological disorders [66]. Although certain studies have limitations, either in the design or number of participants, the majority point to an association between symptoms and DAO deficiency, establishing a general trend that supports the key role of DAO in the etiology of these disorders. A DAO deficiency that predisposes a population subgroup to histamine intolerance may have a genetic, pathological or pharmacological origin [1,41].

Regarding the genetic background of histamine intolerance, several studies have analyzed in depth the polymorphisms in genes encoding the enzymes L-histidine decarboxylase, DAO and HNMT, as well as the different histamine receptors. More than 50 nonsynonymous single-nucleotide polymorphisms (SNPs) in the DAO-encoding gene have been identified, some of which can produce a protein with altered activity and lead to symptoms of histamine intolerance [67,68,69,70,71,72]. Specifically, the most relevant SNPs affecting DAO enzyme functionality in Caucasian individuals are rs10156191, rs1049742, rs2268999 and especially rs1049793 [69,71]. On the other hand, an SNP in the promoter region of the gene has also been identified that causes a lower transcriptional activity of the DAO-encoding gene (rs2052129), as well as several genetic variations responsible for enzyme deficiency in people of Asian or African origin (rs45558339 and rs35070995, respectively) [67,72]. In most cases, the effect of these genetic variations on DAO functionality is through changes in enzyme kinetics, the resulting increase in K_M_ causing a reduction in the rate of histamine degradation [69]. In parallel, three SNPs have been identified as being responsible for enhanced DAO enzyme activity (rs2071514, rs1049748 and rs2071517) [72]. There is also evidence of DAO mutations in patients with certain cardiovascular, gastrointestinal and nervous system pathologies, although with contradictory results regarding positive/negative effects [68].

DAO deficiency can also be an acquired condition, caused by certain pathologies or interaction with drugs. Several inflammatory bowel pathologies affecting mucosal integrity are known to result in impaired DAO activity, the degree of which can be correlated with the severity of mucosal damage [73,74,75]. Thus, DAO activity has been proposed as a marker of integrity of the intestinal mucosa. Miyoshi et al. demonstrated that DAO activity can be a useful predictor of intestinal mucosal damage in patients receiving chemotherapy [76]. Additionally, DAO deficiency has also been linked to certain functional gastrointestinal disorders, such as carbohydrate malabsorption and nonceliac gluten sensitivity (NCGS) [63,73,77,78,79]. Enko et al. found that a concomitant reduction in DAO and lactase enzyme activities could be a consequence of mucosal damage in the small intestine due to gastrointestinal disorders (e.g., gastroenteritis, irritable bowel syndrome, short bowel syndrome and gastrointestinal surgery) [73]. Moreover, patients with lactose intolerance and plasma DAO deficit showed higher end-expiratory H_2_ levels and the appearance of more symptoms during the H_2_ breath test in comparison with lactose-intolerant individuals with normal DAO activity [79]. More recently, two works have suggested a potential relationship between a reduced DAO activity and the presence of NCGS. Schnedl et al. based this relationship on the broad parallelism between the symptomatology of NCGS and histamine intolerance, while the pilot study conducted by Griauzdaite et al. reported a strong association between reduced DAO activity and the presence of NCGS, although with a reduced number of patients [77,78]. In fact, Griauzdaite et al. found out that nine of 10 patients with NCGS had decreased serum DAO activity levels [78]. This recently indicated relationship between both disorders, NCGS and histamine intolerance, should be further explored as it may be of interest for the correct clinical management of affected patients.

Finally, DAO deficiency can be a temporary and reversible condition, caused by the inhibitory effect of substances such as biogenic amines and alcohol, as discussed above, as well as several widely used drugs (Table 2) [1,10]. It has been estimated that approximately 20% of the European population regularly take DAO-inhibiting drugs, which significantly increases the number of people susceptible to the adverse effects of dietary histamine [28]. In vitro experimental results show a potent inhibitory effect (greater than 90%) of chloroquine, a historical antimalarial active ingredient, and clavulanic acid, a β-lactam antibiotic widely used in combination with amoxicillin [80]. A significant inhibition of the enzymatic activity has also been observed with the antihypertensive drug verapamil and the histamine H2 receptor antagonist cimetidine, although the clinical use of the latter is currently anecdotal [23,80]. Other substances have also shown an inhibitory effect, albeit to a lesser extent (Table 2) [23,80,81]. In most cases, the structural similarity of the cited drugs with histamine could explain their potential to bind to the active site of DAO and reduce its enzymatic activity [23]. Along the same lines, substances with an inhibitory effect on other enzymes involved in any of the metabolic pathways of histamine in the body (i.e., HNMT, ALDH and MAO) may act as a trigger of histamine hypersensitivity [82].

### 5.2. Prevalence of DAO Deficit in Persons with Symptoms Related to Histamine Intolerance

Several studies have evaluated the prevalence of DAO deficit in plasma of individuals with symptoms of histamine intolerance and/or diagnosis with certain chronic disorders.

Mušič et al. found DAO deficiency in 80% of 316 adult patients showing various symptoms associated with histamine intolerance (e.g., urticaria, pruritus, diarrhea, abdominal pain, vomiting, constipation, cough, rhinitis and headache), as well as significantly lower plasma DAO activity compared to the control group [83]. Similarly, in a retrospective study, Manzotti et al. evaluated DAO activity in 14 patients with a confirmed diagnosis of histamine intolerance who showed mainly gastrointestinal and dermatological symptoms, but also headaches [84]. In this case, patients showed a high prevalence of DAO deficit (71%) and a significantly lower mean DAO activity compared to healthy volunteers. A lower percentage of DAO deficiency in histamine-intolerant patients (24%) was reported by Pinzer et al. [63]. Those patients featured elevated histamine levels and constantly reduced DAO activities throughout the day.

In a study focused only on headache symptoms, Steinbrecher and Jarisch reported DAO deficiency in 23 of 27 patients (85%) [85]. In parallel, the authors described a significant increase in DAO activity after patients followed a low-histamine diet for four weeks, along with a remission or reduction in frequency of headaches in almost 90% of individuals. More recently, Izquierdo et al. studied the prevalence of DAO deficit in 137 patients diagnosed with a confirmed migraine diagnosis and in a control group of 61 nonmigraine individuals [66]. In this study, a high prevalence of DAO deficiency was observed in the migraine group (87%) and with a mean DAO activity significantly lower in comparison with that obtained from control volunteers. However, the prevalence of DAO deficiency in the control population amounted up to 44%, which was attributed to the fact that certain individuals could present other symptoms associated with histamine intolerance or DAO deficiency other than migraines. Another study with 44 migraine patients reported a 60% prevalence of DAO deficiency and a significant copresence of certain gastrointestinal disorders, such as celiac disease and NCGS [78].

In the field of dermatological symptomatology, several studies have monitored plasma DAO activity in patients with eczema, chronic idiopathic urticaria and atopic dermatitis. Overall, the reported prevalence of DAO deficiency ranges from 19 to 57%, with the exception of the study by Worm et al., who did not detect statistically significant differences in plasma DAO activity between control patients and those with atopic dermatitis [86,87,88,89].

Finally, regarding gastrointestinal symptoms, Honzawa et al. assessed the clinical significance of plasma DAO activity levels in 98 patients suffering inflammatory bowel disease [90]. This study showed that DAO activity in blood was significantly lower in patients with Crohn’s disease and ulcerative colitis compared to the control population, suggesting its potential importance as a marker of intestinal permeability. In a pediatric population under 15 years of age, Rosell-Camps et al. determined DAO deficiency in 88% of patients with abdominal pain, diarrhea and vomiting [91]. In contrast, in a more recent study by a group of Austrian researchers, DAO deficiency was found in only 8% of 394 children with chronic abdominal pain [92].

To date, little data is available on the prevalence of this enzymatic deficiency related to gender, and it is inconclusive. Klockler et al. found no differences in plasma DAO activity between men and women, although the number of individuals considered was scarce (n = 28) [93]. Likewise, the study performed by Izquierdo et al. reported similar percentages of DAO deficiency in migraine-suffering women (83%) and men (90%) [66]. On the contrary, García-Martín et al. did describe differences in plasma DAO activity by gender, with the prevalence of this enzyme deficiency being higher in women [94]. Significant fluctuations in DAO activity values have also been reported in women associated with different stages of the menstrual cycle [94,95].

One factor that could explain the discordance among the prevalence data of DAO deficit in patients with disorders associated with histamine intolerance is that the parameter considered in all of them was serum DAO activity, which, a priori, would not reflect an enzymatic deficiency derived from certain intestinal pathologies. Overall, in spite of the varying percentages in DAO deficiency, the currently available studies seem to indicate an etiological relationship between DAO deficiency and certain symptoms or disorders related to histamine intolerance. Nevertheless, more studies are needed to assess the clinical significance of the determination of plasma DAO activity, as well as to develop new diagnostic methods aimed at identifying individuals with histamine intolerance due to DAO deficiency.

### 5.3. Diagnosis of Histamine Intolerance

**Figure 7:**
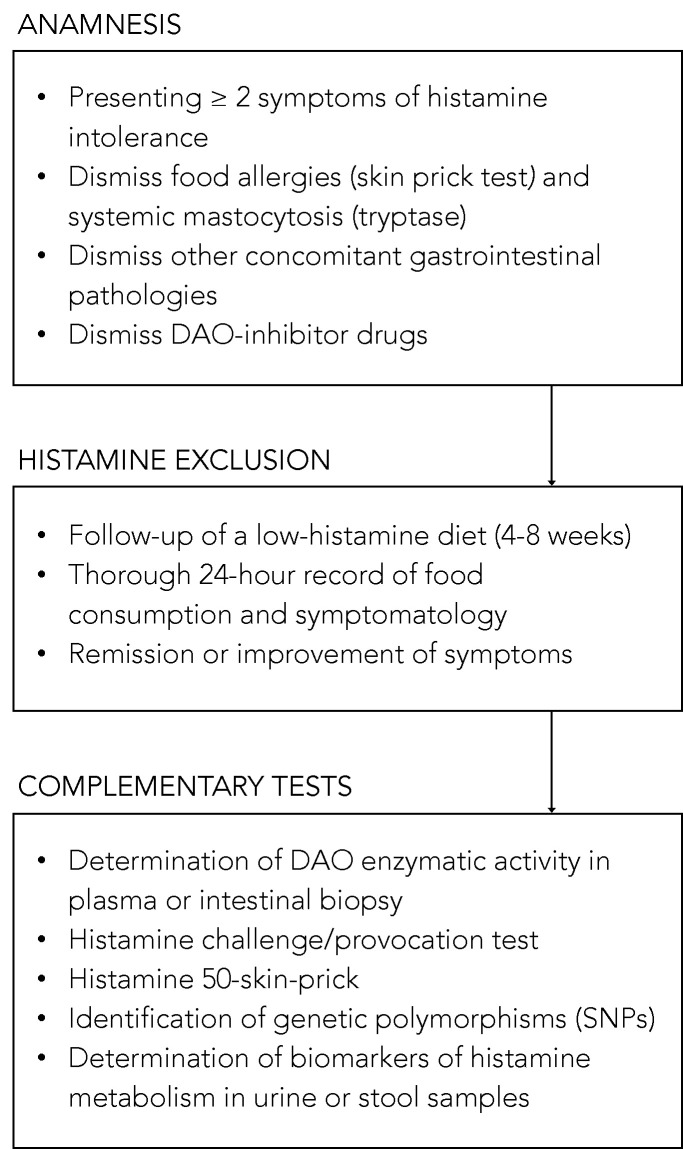
Summary of the described approaches to the diagnosis of histamine intolerance. SNPs: single-nucleotide polymorphisms.

Despite significant advances in the understanding of histamine intolerance, reaching a consensus on a diagnostic algorithm remains a pending challenge. The nonspecificity of symptoms and lack of validated diagnostic tools prompts many affected individuals to go “doctor shopping”; that is, to consult several medical specialists in search of an explanation and solution for their varied symptomatology [13,63]. In the absence of a consensual and clinically validated diagnosis, Figure 7 shows a schematic summary of the diagnostic algorithm for histamine intolerance based on the available scientific evidence reviewed below.

The combination of diagnostic criteria currently in use includes the appearance of typical clinical manifestations and the exclusion of other related disorders [10,13,54]. All the authors who have proposed a diagnostic algorithm for histamine intolerance emphasize the need to initially rule out other potential causes of symptoms associated with an increase in plasma histamine [10,13,54]. For this purpose, it is advisable to carry out an intradermal skin allergy test (i.e., skin prick test) to discard IgE sensitization caused by food allergy, and to measure plasma tryptase to exclude an underlying systemic mastocytosis [10]. It is also important to know whether the patient is taking any medication with a possible inhibitory effect on DAO activity [55]. If these conditions are negative, the appearance of two or more typical symptoms of histamine intolerance and their improvement or remission after the following of a low-histamine diet (i.e., a diet excluding foods that, a priori, contain high histamine levels) will confirm the diagnosis of histamine intolerance [10,54,96,97]. In the diet follow-up, a thorough 24-h record of all the foods consumed and symptoms experienced is recommended in order to establish a relationship, if any, between a food and the onset of symptoms [10,13]. The duration of the low-histamine diet to confirm the diagnosis is not clearly stipulated, although some studies suggest a period of 4 to 8 weeks [54,97]. In addition to the diet, testing the effect of antihistamine treatment on symptoms has also been proposed, although its usefulness once dietary histamine is removed is unclear [10,54].

Once it has been established that dietary histamine is responsible for the intolerance-associated symptoms, the diagnosis of this disorder is virtually confirmed. A range of nonvalidated complementary tests have also been proposed by several authors with the aim of obtaining a marker to confirm the diagnosis [97]. However, it has to be taken into account that not all of the tests consider the different origins of DAO deficiency (i.e., genetic, pathological or pharmacological). Thus, a genetic origin would lead to a reduction of the DAO enzymatic activity in the whole organism. Likewise, the pharmacological blockade of DAO would take place in all tissues where the drug is distributed after entering the systemic circulation, although in a punctual manner upon the substance’s introduction. Lastly, the scope of a DAO deficit due to intestinal pathologies would be limited to the local intestinal environment.

Due to the genetic background of DAO deficiency, one of the strategies for the diagnosis could be the determination of genetic polymorphisms (SNPs) that characterize the population as genetically susceptible to histamine [54]. Currently, there is already the possibility of performing a noninvasive genetic analysis capable of identifying three of the SNPs associated with reduced DAO activity (i.e., rs10156191, rs1049742 and rs1049793) from a sample of the oral mucosa, although evidence-based studies on the diagnosis potential of this test are still needed. It is important to note that this test will only reflect the existence of a genetic DAO deficiency.

The most studied, and possibly also the most controversial, is the determination of plasma DAO activity. This analytical test consists of measuring the amount of histamine degraded in a blood sample in a given time interval. Two types of commercial testing kits are currently available on the market, one consisting of an ELISA-type immunoassay, and the other a radioimmunoassay using radioactively labeled putrescine [83,84]. The evidence for the validity of blood DAO activity measurements for the diagnosis of histamine intolerance is neither abundant nor conclusive. Some studies have proposed that determining blood DAO activity may be helpful in identifying subjects with symptoms associated with histamine intolerance [63,83,84]. In contrast, three studies did not find a significant relationship between the clinical history of patients with typical symptoms of histamine intolerance and blood DAO activity values, concluding that this technique cannot be recommended as a diagnostic tool in routine clinical practice until studies have validated its effectiveness [98,99,100]. Moreover, the work performed by Schnoor et al. also reported a high interassay variation in DAO activity values that made the proper classification of histamine-intolerant subjects impossible [100]. This controversy is described in a joint article published in 2017 by the German and Swiss allergology societies, which emphasizes the need for more research before giving plasma DAO activity a definitive diagnostic value for histamine intolerance [97].

A variant of the intradermal skin allergy test called the histamine 50-skin-prick test was also proposed by Kofler et al. to diagnose histamine intolerance [101]. In this technique, the results were read after 50 min (as opposed to the usual 20 min) and showed that, although the size of the wheal did not differ between the histamine intolerant and control groups, the time course was significantly different. Patients with symptoms of intolerance showed a delayed remission of the wheal induced by cutaneous administration of histamine, signaling a reduced degradation ability. The same results were obtained in a study recently published by Wagner et al., who re-evaluated this skin test as a diagnostic tool of histamine intolerance, also observing a correlation between the delay in wheal disappearance and a lower plasma DAO activity [102].

Both the determination of plasma DAO activity and the histamine 50-skin-prick test could be suitable tests to identify a DAO deficiency from genetic or pharmacological origin, but they would not be useful to determine a deficit secondary to certain intestinal diseases.

On the contrary, there are certain alternatives, such as the intestinal biopsy, the histamine provocation test or the histamine metabolomics in urine, that could make it possible to diagnose histamine intolerance due to DAO deficiency without excluding any of the possible etiological causes.

The measurement of intestinal DAO activity by a colon biopsy during endoscopic procedures has been studied as a possible diagnostic marker. The few available studies have shown a reduced intestinal DAO catabolic activity in patients with recurrent urticaria, food allergy and colon adenoma, accompanied by an increase in histamine levels [103,104,105,106]. Although this test has interesting diagnostic potential, more studies are needed to validate its clinical significance and its relationship with the symptoms of histamine intolerance [97]. If proven, this diagnostic test would be very adequate since this disorder originates from a reduced ability of the intestinal DAO enzyme to cope with dietary histamine.

Histamine challenge/provocation test has also been proposed by some authors as a diagnostic tool for intolerance, which would, at the same time, establish the individual tolerance threshold. This double-blind, placebo-controlled test involves oral administration of histamine and requires patient medical supervision and hospitalization. In the study by Wöhrl et al., half of the healthy volunteers developed symptoms after the administration of a solution containing 75 mg of histamine [107]. In contrast, the results of a multicenter study by Komericki et al. using the same oral dose of histamine indicated the challenge test was unreliable for diagnosing histamine intolerance due to a lack of intraindividual reproducibility of symptoms after two different provocation tests [108]. The application of this procedure is still limited because of the risk of serious adverse side effects and the absence of a standardized dose of histamine and properly established protocol [97].

Finally, in recent years, efforts have been made to identify a noninvasive marker to establish a solid and clinically irrefutable diagnostic criterion for histamine intolerance due to DAO deficiency. Currently, the application of metabolomics as a tool for the identification of biomarkers of histamine metabolism in urine is also being challenged as a possible new diagnostic strategy [11]. The hypothesis is that individuals with histamine intolerance could have a different excretion profile of histamine and its metabolites in urine than normal individuals. For this purpose, Comas-Basté et al. have recently proposed a chromatographic approach that allows for determining in a fast and unequivocal manner the urinary levels of histamine and its methylated metabolite, methylhistamine [11]. It is still necessary to validate the potential diagnostic utility of this approach in patients with histamine intolerance, as well as complementing the excretion profile with other histamine metabolites to obtain a more accurate image of the possible alterations produced in this intolerance.

### 5.4. Treatment Approaches to Histamine Intolerance

Currently, the main strategy to avoid the symptoms of histamine intolerance is to follow a low-histamine diet. Supplementation with exogenous DAO has recently been postulated as a complementary treatment to enhance dietary histamine degradation in intolerant individuals who have a deficiency of this enzyme in the intestine [109,110].

#### 5.4.1. Low-Histamine Diet

A low-histamine or histamine-free diet has been proposed as the main strategy for the preventive treatment of histamine intolerance [10,54,82,111]. Conceptually, these diets exclude a number of foods that patients associate with the onset of symptoms, primarily those that may contain high levels of histamine [82]. However, there is no a single dietary recommendation of a low-histamine diet. As it may be seen in Table 3, there is no coincidence in all the foods excluded in the different low-histamine diets found in the literature [10,87,91,112,113,114,115,116,117,118].

Histamine is widely distributed in different food categories and in highly variable concentrations, as its accumulation is influenced by multiple factors [3,119]. In fresh foods such as fish and meat, and in some derived products, the presence of histamine is due to a lack of freshness or an inadequately hygienic quality of raw materials and/or production processes [31]. For this reason, meat and fish can be consumed in the framework of a low-histamine diet, as long as their freshness is ensured. In contrast, fermented products are systematically excluded, due to a high probability of containing histamine [31]. Other foods such as spinach, eggplant and tomatoes should also be avoided for the same reason. In general, all these abovementioned foods are unanimously eliminated in most published low-histamine diets (Table 3).

On the other hand, there are certain foods that a priori do not contain histamine, but that patients associate with the appearance of symptoms. For these foods, there is much more variability when it comes to their exclusion from low-histamine diets (Table 3). The exclusion of foods could be based on their content of other biogenic amines, such as putrescine and cadaverine, which act as competitive substrates for DAO and may therefore inhibit intestinal degradation of histamine if present in significant quantities [1,82]. Thus, the onset of symptoms after the consumption of citrus fruits, mushrooms, soybeans, bananas and nuts may be due to high levels of other amines, specially putrescine [82]. These diets may also exclude certain foods free of histamine and with low enough concentrations of other amines to justify their exclusion. This is the case, for example, for papayas, kiwis, strawberries, pineapples and plums, which have been reported to trigger the release of endogenous histamine, although the mechanism responsible has not yet been elucidated [8,13].

The effectiveness of a low-histamine diet has been demonstrated in clinical studies, which report favorable results in terms of improvement or total remission of symptoms frequently associated with histamine intolerance and DAO deficiency (Table 4). As shown in Table 4, over the past three decades, various clinical studies have assessed the effect of a low-histamine diet on the evolution of various symptoms, mainly dermatological, gastrointestinal and neurological, including cases with more than one type. Although most studies have involved only a small group of patients (a mean of 38 per study, with a minimum of 10 and maximum of 157), they report an efficacy rate for the diet ranging from 33% to 100%. Specifically, 10 of the 13 studies reviewed found an improvement in symptoms in more than 50% of patients who followed the diet; two studies show success rates of less than 50% (33% and 46%), and only one did not observe any beneficial effects (Table 4). Most of the studies involved patients with dermatological symptoms, primarily chronic idiopathic urticaria, atopic dermatitis and eczema. In this field, a recent systematic literature review included a total of 1668 patients with chronic urticaria undergoing different exclusion diets, including low-histamine, pseudoallergen-free (i.e., without preservatives and artificial colors present in processed foods or aromatic compounds from certain natural products) and fish exclusion diets [120]. Overall, following any of the exclusion diets resulted in the total or partial remission of symptoms in 4.9% and 37.5% of patients, respectively. A low-histamine diet for an average of 3 weeks resulted in one of the highest remission rates. Despite the promising results of a low-histamine diet for the treatment of dermatological conditions, scientific societies of dermatology still consider this exclusion diet of unproven utility pending randomized, double-blind, placebo-controlled clinical trials to confirm its effectiveness [121].

In general, the duration of the dietary treatment considered in the different clinical studies ranges from 3 to 4 weeks, and no positive relationship could be established between a longer duration and the success rate in symptom remission (Table 4). As may also be seen in this table, some studies have also assessed the effect of diet on other variables, such as plasma histamine levels or plasma DAO activity [83,85,86,87,112,122,123]. Regarding DAO activity, the studies published by Steinbrecher et al., Maintz et al., Mušič et al. and Lackner et al. all point out an increase in plasma enzymatic activity in more than 50% of patients after the dietary intervention, although no explanatory hypothesis has been yet suggested [83,85,86,123]. In contrast, Guida et al., Wagner et al. and Son et al. reported no changes in serum DAO activity [87,112,122]. The inconsistency of these data highlights the need to develop more research in this specific field before conclusions can be drawn.

**Table 4:** Clinical studies on the efficacy of a low-histamine diet for the treatment of symptoms of histamine intolerance.

Design and Outcomes of the Study	Number of Patients and Symptoms	Duration	Percentage of Patients with Improvement in the Study Outcomes	Reference
Prospective study with evaluation of the evolution of the symptomatology	28 patients with chronic headache and 17 with other dermatological and respiratory symptoms	4 weeks	68% reduction in chronic headache and 82% reduction in other symptoms	[124]
Prospective study with evaluation of the evolution of symptoms, plasma histamine levels and DAO activity	10 patients with chronic idiopathic urticaria and 19 control individuals	3 weeks	100% reduction in symptoms, 100% reduction in plasma histamine and no changes in DAO activity	[122]
Prospective study with evaluation of the evolution of symptoms, plasma histamine levels and DAO activity	35 patients with headache and other symptoms (urticaria, arrhythmia, diarrhea and asthma)	4 weeks	77% reduction in symptoms, 73% increase in DAO activity and no changes in plasma histamine levels	[85]
Prospective study with evaluation of the evolution of symptoms and DAO activity (in five of the patients)	17 patients with DAO deficiency, atopic eczema and other symptoms (headache, flushing and gastrointestinal symptoms)	2 weeks	100% reduction in symptoms and 60% (three out of five) increase in DAO activity	[86]
Prospective study with evaluation of the evolution of symptoms and the use of antihistamine drugs	13 patients with chronic idiopathic urticaria and 35 control patients (without diet)	4 weeks	Lack of improvement in symptoms and no changes in the use of antihistamines	[125]
Prospective study with evaluation of the evolution of the symptomatology	36 patients with atopic dermatitis and 19 control individuals	2 weeks	33% reduction in symptoms	[88]
Prospective study with evaluation of the evolution of the symptomatology and DAO activity	20 patients with DAO deficiency and dermatological, gastrointestinal and respiratory symptoms	6–12 months	100% reduction in symptoms and 100% increase in DAO activity	[83]
Retrospective study with evaluation of the evolution of the symptomatology	16 pediatric patients with diffuse abdominal pain, diarrhea, headache, vomiting and rash	4 weeks	100% reduction of symptoms	[91]
Prospective study with evaluation of the evolution of the symptomatology	16 pediatric patients with chronic abdominal pain and DAO deficiency	4 weeks	88% reduction of symptoms	[92]
Retrospective study with evaluation of the evolution of the symptomatology	157 patients with chronic idiopathic urticaria	4 weeks	46% reduction of symptoms	[126]
Prospective study with evaluation of the evolution of the symptomatology and DAO activity	56 patients with chronic idiopathic urticaria and gastrointestinal symptoms	3 weeks	75% reduction in symptoms and no changes in DAO activity	[87]
Prospective study with evaluation of the evolution of symptoms, plasma histamine levels and DAO activity	22 patients with chronic idiopathic urticaria	4 weeks	100% reduction in symptoms, 100% reduction in plasma histamine levels and no changes in DAO activity	[112]
Retrospective study with evaluation of the evolution of the symptomatology and DAO activity	63 patients with gastrointestinal symptoms	7–18 months	79% reduction in symptoms and 52% increase in DAO activity	[123]

#### 5.4.2. Exogenous DAO Supplementation

Similar to the current treatment for lactose intolerance, the possibility of oral supplementation with exogenous DAO has been proposed by several authors to facilitate dietary histamine degradation [13,127]. Improving intestinal DAO activity would allow a less restrictive diet, which could include foods with a tolerable dose of histamine [10,61]. In this context, in the update of the official list of novel foods in 2017, the European Commission gave the green light to the marketing of a DAO supplement as food supplement or as food for special medical purposes [128]. Specifically, European regulations authorize the formulation of porcine kidney protein extract with an enteric coating to ensure its integrity during its passage through the gastric environment [128]. In this specific regulation, the minimum DAO enzymatic capacity required for the supplement is determined through a radio extraction assay (REA). This technique, based on the radioactive labeling of putrescine and the scintillation counting of its consumption, is advantageous in terms of rapidity and sensitivity, but it was mainly conceived to be applicable to serum samples. Comas-Basté et al. have recently developed a rapid and reliable methodology through ultra-high performance liquid chromatography and fluorimetric detection (UHPLC-FL) for the in vitro determination of DAO activity specifically for the analysis of unpurified complex matrixes, such as porcine kidney extract and DAO supplements [129]. This methodological approach is based in the direct determination of histamine degradation and overcomes certain drawbacks in terms of matrix interferences and handling of radioactive materials.

Porcine kidneys are the main source of DAO enzyme, according to the literature. Several studies have demonstrated the capacity of this product to degrade histamine and other biogenic amines in vitro [57,129,130,131,132,133]. A wide variability of the DAO capacity of porcine kidney extracts has been reported (with values ranging from 0.1 to more than 100 mU/mg), depending on the purification grade applied to the matrix and/or the amine compound used as the reaction substrate [57,129,130,131,132,133]. In fact, many of these works sought the selective purification of the DAO enzyme to design biosensors for the biorecognition of biogenic amines as indicators of freshness in foods [134]. More recently, two studies have been published specifically focused in investigating the in vitro DAO activity of porcine kidney protein extract expressly used as active ingredient to formulate food supplements for the preventive treatment of histamine intolerance [129,130]. Comas-Basté et al. studied the in vitro enzymatic activity of 13 different production batches of porcine kidney protein extract used in the elaboration of food supplements, reporting a low influence of the raw material (porcine kidney) on the DAO activity of the extract, with a mean value of 0.23 ± 0.01 mU/mg [129]. Later, Kettner et al. obtained a porcine kidney crude extract with an in vitro DAO activity of 0.5 ± 0.06 mU/mg, and described a 10-fold increase of this enzymatic activity through the application of several consecutive purification steps [130].

Regarding the food supplement, divergent results have also been reported, since while certain authors discard its enzymatic capacity, DAO activity values ranging from 0.04 to 0.20 mU/mg have also been described for different commercial products available in the market [129,130]. Overall, these works coincide in the need to identify alternative sources to porcine kidney DAO for exogenous supplementation in histamine-intolerant individuals.

A higher catalytic capacity of DAO enzymes of plant origin in degrading certain amino substrates has been described by some authors in comparison with those of animal origin [129,135,136,137]. Specifically, the germinated sprouts of certain edible legumes have been pointed out as interesting sources of DAO enzyme. Germination is a physiological process that has been described as capable of increasing the DAO enzymatic capacity of sprouts by up to 250 times compared to ungerminated seeds [61,138]. The increased presence of DAO enzyme in legume sprouts could be associated with the importance of hydrogen peroxide, a byproduct of the deamination reaction, in the cell wall structuring, lignification and mobilization of seed reserves during germination [139,140,141,142]. In fact, it has been demonstrated that the germination of legume seeds for a period of 6–8 days in darkness provides the optimal environment to maximize the DAO activity of this plant-origin matrix [61,138,143]. From a commercial point of view, having a plant source of this enzyme would expand the target of this novel food for the vegetarian/vegan population, as well as those with religious restrictions on the consumption of pork products. In addition, obtaining DAO enzyme from legumes would be a practice in accordance with the current call for action of the Sustainable Development Goals.

Nowadays, only five published intervention studies (four from the last five years) have tested the clinical efficacy of exogenous DAO supplementation in patients with symptoms of histamine intolerance (Table 5). Although there is some variability, the available research points to the effectiveness of DAO supplements in reducing the appearance and intensity of symptoms. However, it is difficult to compare the different studies, since they differ in the design, the enzyme dosage, the intervention time and the measurement of efficacy outcomes. Komericki et al., Manzotti et al. and Schnedl et al. assayed the efficacy of DAO supplementation in patients with diverse symptoms associated with histamine intolerance (gastrointestinal, cardiovascular, respiratory and dermatological and/or neurological complaints) [84,108,109]. All three studies reported an important improvement in the intensity or frequency of symptoms, although they involved a small study population (14, 28 and 39 patients) and/or a reduced intervention time (from two to four weeks). Moreover, Schnedl et al. also evaluated the changes in plasmatic DAO activity, reporting a slight increase in 61% of patients during the intervention, which the authors linked to a possible improvement in the integrity of the intestinal mucosa due to the supplementation [109].

The clinical trials developed by Yacoub et al. and Izquierdo-Casas et al. were focused on a single disorder related to histamine intolerance (chronic spontaneous urticaria and migraine, respectively) [110,144]. Yacoub et al. considered 20 patients with chronic spontaneous urticaria who showed a significant reduction in the severity of the complaint according to the Urticaria Activity Score (UAS-7) [144]. Regarding migraines, the randomized double-blind clinical trial conducted by Izquierdo et al. considered a larger number of patients (100 patients) and obtained a statistically significant decrease in the duration of pain attacks with no recorded adverse side effects [110]. However, the authors did not find statistical differences when considering other research outputs, such as the frequency and intensity of pain.

Overall, despite the promising results, more ambitious clinical studies with a rigorous experimental design, longer treatment periods and properly sized samples are essential to establish the clinical efficacy of this treatment.

## 6. Conclusions and Perspectives

Histamine intolerance is currently a clinical entity of increasing interest, which can appear due to the intake of histamine from foods, mainly caused by a deficiency of the DAO enzyme at the intestinal level. Novel knowledge and studies of histamine intolerance have helped clarify many of the uncertainties that were classically associated with histamine intoxication. Etiologically, various SNPs have been identified in the gene encoding the DAO enzyme related to lower enzyme activity. Moreover, certain inflammatory bowel diseases that limit enzyme secretion or some DAO-inhibiting drugs have also been identified as possible causes of DAO deficiency. This intolerance manifests through a plethora of nonspecific gastrointestinal and extraintestinal symptoms.

The diagnosis of histamine intolerance is usually performed after ruling out allergic symptoms and by the presence of at least two clinical manifestations and their improvement or remission after following a low-histamine diet. Various complementary tests are currently being proposed to improve the diagnosis of this intolerance based, among others, on determining the DAO activity in blood or intestinal biopsy samples or on identifying genetic or metabolic urinary markers by noninvasive techniques.

The clinical management is carried out mainly through the follow-up of a low-histamine diet, although there is no consensus on the list of foods to be excluded. Even so, there are different clinical studies that show the efficacy of this dietary intervention in improving the quality of life of patients with symptoms of histamine intolerance. Oral supplementation with exogenous DAO enzyme from porcine kidney is also being used to enhance the intestinal capacity to degrade dietary histamine. Although few works have assayed the clinical efficacy of this preventive treatment, promising results have been obtained so far. Research is currently also being made to identify new sources of DAO enzyme, especially of plant origin, due to its higher catalytic capacity and other potential productive and commercial advantages.

In this context, it is necessary to keep promoting the multidisciplinary study of this disorder, both from basic (i.e., analytical chemistry, food science, physiology and biochemistry) and clinically applied research, meant to increase the scientific base and the currently available diagnostic and treatment strategies for histamine intolerance.

## Figures and Tables

**Table 1:** Histamine content in different food categories. Adapted from [31].

Food		Histamine Content (mg/kg)			
	n	Mean (SD)	Median	Minimum	Maximum
Fruits, vegetables and plant-based products					
Fruits	136	0.07 (0.20)	ND	ND	2.51
Nuts	41	0.45 (1.23)	ND	ND	11.86
Vegetables	98	2.82 (7.43)	ND	ND	69.72
Legumes	11	ND	ND	ND	ND
Cereals	28	0.12 (0.33)	ND	ND	0.89
Chocolate	25	0.58 (0.44)	0.17	0.16	0.56
Spices	12	ND	ND	ND	ND
Alcoholic beverages					
Beer	176	1.23 (2.47)	0.70	ND	21.60
White wine	83	1.24 (1.69)	0.45	0.10	13.00
Red wine	260	3.81 (3.51)	1.90	0.09	55.00
Fish and seafood products					
Fresh fish	136	0.79 (0.71)	ND	ND	36.55
Canned fish	96	14.42 (16.03)	5.93	ND	657.05
Semipreserved fish	49	3.48 (3.37)	2.18	ND	34.90
Meat and meat products					
Fresh meat	6	ND	ND	ND	ND
Cooked meat	48	0.30 (0.26)	ND	ND	4.80
Cured meat	23	12.98 (37.64)	0.80	ND	150.00
Dry-fermented sausages	209	32.15 (14.22)	8.03	ND	357.70
Dairy products					
Unripened cheese	20	ND	ND	ND	ND
Raw milk cheese	20	59.37 (106.74)	18.38	ND	389.86
Pasteurized milk cheese	20	18.05 (38.23)	4.59	ND	162.03

ND: not detected.

**Table 2:** Active ingredients with an experimentally demonstrated inhibitory effect on the DAO enzyme [23,28,80,81].

Active Ingredient	Indication
Chloroquine	Antimalarial
Clavulanic acid	Antibiotic
Colistimethate	Antibiotic
Cefuroxime	Antibiotic
Verapamil	Antihypertensive
Clonidine	Antihypertensive
Dihydralazine	Antihypertensive
Pentamidine	Antiprotozoal
Isoniazid	Antituberculous
Metamizole	Analgesic
Diclofenac	Analgesic and anti-inflammatory
Acetylcysteine	Mucoactive
Amitriptyline	Antidepressant
Metoclopramide	Antiemetic
Suxamethonium	Muscle relaxant
Cimetidine	Antihistamine (H2 antagonist)
Prometazina	Antihistamine (H1 antagonist)
Ascorbic acid	Vitamin C
Thiamine	Vitamin B1

**Table 3:** Foods excluded in the different low-histamine diets found in the literature [10,87,91,112,113,114,115,116,117,118].

Foods Excluded by Low-Histamine Diets		
<20% *	20–60% *	>60% *
Milk	Shellfish	Cured and semicured cheese
Lentils	Eggs	Grated cheese
Chickpeas	Fermented soy derivatives	Oily fish
Soybeans	Eggplant	Canned and semipreserved oily fish derivatives
Mushrooms	Avocado	Dry-fermented meat products
	Banana	Spinach
	Kiwi	Tomatoes
	Pineapple	Fermented cabbage
	Plum	Citrus
	Nuts	Strawberries
	Chocolate	Wine
		Beer

* Percentage of low-histamine diets from the literature that exclude each foodstuff.

**Table 5:** Studies on the efficacy of DAO enzyme supplementation for the treatment of symptoms of histamine intolerance.

Design	Number of Patients and Symptoms	Duration of DAO Supplementation	Efficacy Outcomes	Reference
Randomized, double-blind, placebo-controlled, crossover provocation study using histamine-containing and histamine-free tea in combination with DAO capsules or placebo	39 patients with histamine intolerance (headache and gastrointestinal and skin complaints)	-	Statistically significant reduction of histamine-associated symptoms compared to placebo	[108]
Retrospective study with evaluation of the clinical response to DAO supplementation	14 patients with diagnosis of histamine intolerance (headache and gastrointestinal, cardiovascular, respiratory and skin complaints)	2 weeks	Reduction of at least one of the reported symptoms in 93% of patients	[84]
Double-blind, placebo-controlled, crossover study	20 patients with chronic spontaneous urticaria	1 month	Significant reduction of 7-Day Urticaria Activity Score (UAS-7) and slight significant reduction of daily antihistamine dose	[144]
Randomized, double-blind, placebo-controlled clinical trial	100 patients with episodic migraine and serum DAO deficit	1 month	Significant decrease in the duration of migraine attacks and decrease in triptans intake	[110]
Open-label interventional pilot study	28 patients with histamine intolerance (gastrointestinal, cardiovascular, respiratory and skin complaints) and reduced serum DAO values	1 month of intervention and 1 month of follow-up	Significant improvement in frequency and intensity of all symptoms. 61% of patients showed slightly increase in serum DAO values. During the follow-up period (without DAO supplementation), the symptoms sum scores increased and DAO levels decreased.	[109]
